# Optimizing stimulation parameters in functional electrical stimulation of denervated muscles: a cross-sectional study

**DOI:** 10.1186/s12984-015-0046-0

**Published:** 2015-06-07

**Authors:** Karin Pieber, Malvina Herceg, Tatjana Paternostro-Sluga, Othmar Schuhfried

**Affiliations:** Department of Physical Medicine and Rehabilitation, Medical University of Vienna, General Hospital of Vienna, Waehringer Guertel 18-20, 1090 Vienna, Austria; Institute of Physical Medicine and Rehabilitation, Donauspital, Vienna, Austria

**Keywords:** Electrical stimulation, Denervation, Chronaxie, Polarity, Pulse duration

## Abstract

**Background:**

To counteract denervation atrophy long-term electrical stimulation with a high number of muscle contractions has to be applied. This may lead to discomfort of the patient and negative side effects like burns. A functional effective muscle contraction induced by the lowest possible stimulation intensity is desirable. In clinical practice a selective stimulation of denervated muscles with triangular pulses is used.

The aim of the study was to evaluate the influence of polarity and pulse duration on the stimulation intensity of triangular pulses in denervated muscles in patients with peripheral nerve lesions.

**Methods:**

Twenty-four patients with denervated extensor digitorum communis muscle and twenty-four patients with denervated tibialis anterior muscle due to peripheral nerve lesions were included. Four different combinations of triangular pulses with various duration and polarity were delivered randomly to the denervated muscles. The threshold intensity to induce a functional effective muscle contraction was noted. One-way within subject ANOVA was used to assess changes in intensity. An alpha level of p less than or equal to 0.05 was the criterion for statistical significance.

**Results:**

Patients with a denervated tibialis anterior muscle presented significant lower intensities inducing a functional effective muscle contraction in favor of the stimulation with a duration of 200 ms and a polarity with the cathode proximally applied. No significant differences could be shown between the different stimulation protocols in case of denervated extensor digitorum communis muscle.

**Conclusions:**

We recommend electrical stimulation of the denervated tibialis anterior muscle with triangular current with a duration of 200 ms and a polarity with the cathode proximally applied.

## Background

Denervation of muscles leads to loss of voluntary and reflex activity, muscle atrophy and changes in muscle excitability [1]. Denervated muscles are different from innervated muscles in their response to electrical stimulations. A direct stimulation of the muscle fiber with a greater electric charge is needed [2]. To selectively stimulate a denervated muscle, a slow rising triangular pulse is required as this muscle has less ability to accommodate [2]. This selective stimulation is necessary to avoid the overstimulation of the innervated muscles in the surroundings and to avoid the stimulation of sensory fibers [3]. The long rise time and the long pulse duration is suitable to stimulate denervated muscle fibers since a denervated muscle has higher chronaxie values. Chronaxie is defined as the duration (in ms) of a rectangular pulse with the two-fold intensity of the rheobase which just reaches the stimulation threshold at which a muscle twitch occurs. Chronaxie values above 1 ms are described as a sign of muscle denervation [4, 5]. To cause a contraction in denervated muscles either rectangular pulses of sufficient duration (30 ms or more) or triangular pulses of long duration (100–500 ms) can be used [2]. But the use of rectangular pulses can result in excessive recruitment of neighboring innervated muscles and can be limited due to patient’s sensation due to stimulation of sensory fibers [3, 6]. In clinical practice, mainly triangular pulses with a pulse duration of 200 ms or 500 ms are used. By the use of more than 100 ms pulse duration, the strength-duration curves (also known as intensity/time curve - I/t-curve) for triangular pulses differs between denervated and innervated muscles with a higher threshold for innervated muscle. Therefore, the selective stimulation of the denervated muscle is possible [3, 7]. The threshold current intensity might be significantly lower when stimulating with 500 ms rather than 200 ms. Because of the positively charged outer surface of the nerve membrane the negatively charged electrode (cathode) is more effective in stimulating excitable tissue than the positively charged electrode (anode) [1, 2]. Therefore, a change of polarity might require a different current intensity to stimulate a muscle. Overall, the stimulation should achieve a moderately strong contraction without unnecessary discomfort for the patient [3] and resemble a normal activity of the motor neuron [8].

Functional electrical stimulation (ES) is one treatment option for denervated muscles to preserve or restore muscle strength and reduce or prevent muscle atrophy due to denervation and disuse [1, 2]. To our knowledge, only few human studies are dealing with this topic [9–13]. These studies included patients suffering from spinal cord injuries [9, 11, 13], lumbosacral plexus avulsion trauma with a lower extremity monoparesis [10] or total peripheral nerve lesions of the median, ulnar or peroneal nerve [12]. They used rectangular pulses with long duration [9, 11–13]. One study used transcutaneous electrical muscle stimulation without information concerning the stimulation parameters [10]. Most of these studies were not recently published [6, 9–12] and were no RCTs [6, 9–11, 13], but case based pilot or longitudinal studies with a small sample size. Only Boonstra et al. [12] used a randomized-controlled design. But the author had to mention the sometimes bad motivation of the patients and therefore not optimal performance of the ES which may have negative impact on the results. The effects of ES in denervated muscles have been discussed controversially concerning maintaining muscle mass until re-innervation and possible delay in neuromuscular recovery [14, 15].

To the best of our knowledge, no human study has evaluated ES with triangular pulses in denervated muscles. Animal experiments with triangular pulses have shown a decreased expression of atrophy genes, but ES did not avert the loss of muscle mass due to denervation [16, 17]. This may be explained by the low number of muscle contractions triggered by ES [18].

In ES of denervated human and animal muscles triangular and rectangular pulses have not been compared yet. Nevertheless, this is an important topic in rehabilitation of peripheral nerve lesions as functional ES with triangular pulses is broadly and successfully used in clinical practice although recommendations are mostly based on clinical books and empirical [1, 2].

No study has evaluated in detail the effect of pulse duration and polarity of triangular pulses in the stimulation of human denervated muscle. Thus, the aim of this study was to evaluate the impact of stimulation parameters such as polarity and pulse duration during functional ES with triangular pulses of denervated muscles in the upper and lower extremity in patients with peripheral nerve lesions on required intensity for an effective muscle contraction.

## Methods

### Participants

Twenty-four patients (9 female/15 male) with denervated extensor digitorum communis muscle (EDC) and twenty-four patients (8 female/16 male) with denervated tibialis anterior muscle (TA) were included in this study. Characteristics of included patients are presented in Table 1.

**Table 1:** Characteristics of the study population

	Extensor digitorum communis muscle (n = 24)	Tibialis anterior muscle (n = 24)
Age (years)	47.2 ± 18.4 (19–78)	51.9 ± 18 (23–81)
Duration of lesion (weeks)	14.5 ± 21.1 (2–100)	13.2 ± 14.1 (2–50)
Strength (MRC scale)	1.0 ± 0.9 (0–3.5)	2.2 ± 1.4 (0–4)
Chronaxie (ms)	16.9 ± 18.9 (0.4–80)	16.2 ± 9.5 (0.7–35)

Data in mean ± SD (range), *ms* milliseconds

### Setup

All examinations were conducted in the electrophysiological laboratory of the Department of Physical Medicine and Rehabilitation of the Medical University of Vienna. Two senior physicians, experienced in nerve conduction velocity studies (NCV), evaluations of chronaxie and functional ES, performed the examinations. The cross-sectional testing were carried out during daily routine of an outpatient department. Patients with a denervated EDC due to a lesion of the radial nerve, plexopathy or radiculopathy or denervated TA due to a lesion of the peroneal nerve, plexopathy or radiculopathy referred to electrophysiological examination and evaluation for long-term ES were included consecutively in this study. Patients with severe impairments of cognition and communication, epilepsy, cardiac pacemakers, edema, skin lesions and younger than 19 years were excluded. The testing was not wearing, the study protocol was approved by the head office of the Department and performed according to the guidelines of good clinical practice, following the principles of the Declaration of Helsinki.

### Procedure (see Fig. 1)

**Fig. 1:**
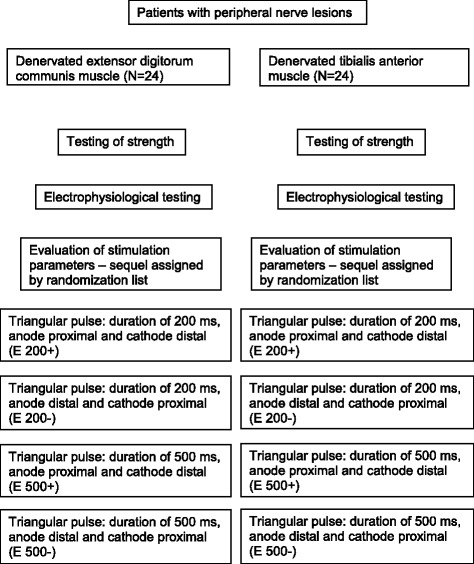
Procedure of testing and stimulation protocol

The peripheral nerve lesion was confirmed by NCV or by evaluating chronaxie values. The time from the onset of the lesion was recorded in weeks. Prior to electrophysiological testing the strength of the EDC and TA was assessed using the Medical Research Council Scale (MRC Scale) which uses the numeral grades 0–5 (0 – no movement, 1 – a flicker of movement is seen or felt in the muscle, 2 – muscle moves the joint when gravity is eliminated, 3 – muscle cannot hold the joint against resistance, but moves the joint fully against gravity, 4 – muscle holds the joint against a combination of gravity and moderate resistance, 5 – normal strength) [19, 20]. Electrophysiological examination for determination of chronaxie was performed with a constant-current electrical stimulator (Endomed 982, Enraf-Nonius, Netherlands) as described by Paternostro-Sluga et al. [5] and NCV with an electro-diagnostic machine (Keypoint; Medtronic, Dantec Medical A/S, Skovlunde, Denmark).

**Fig. 2:**
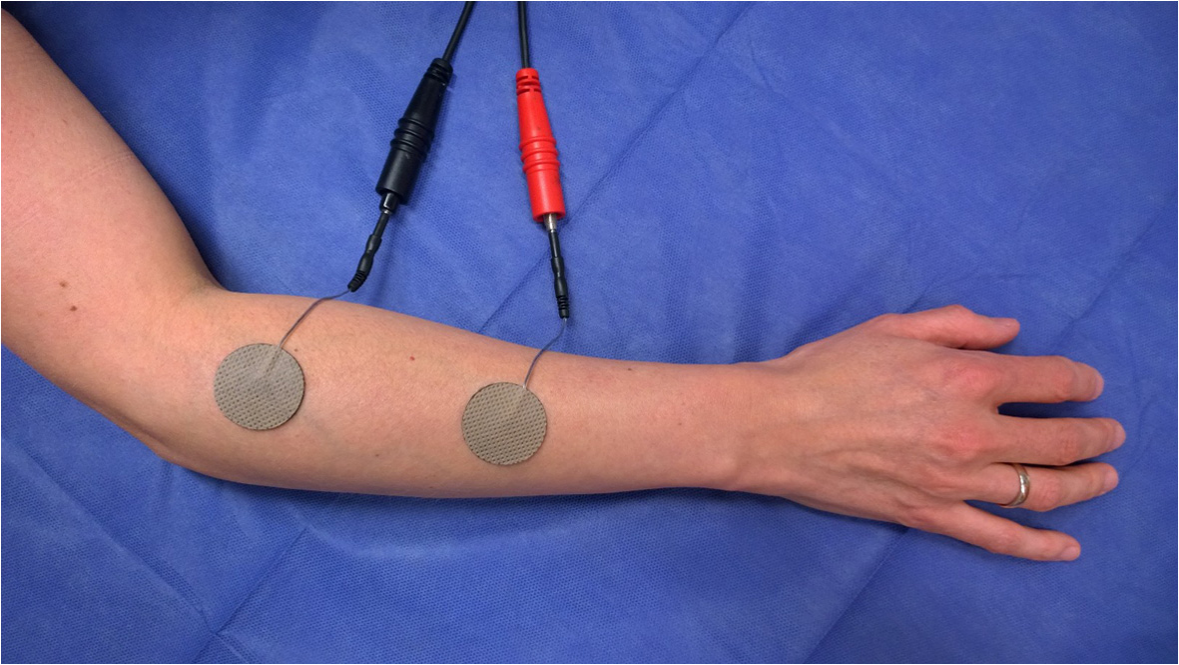
Stimulation of the extensor digitorum communis muscle (EDC). Positioning of electrodes in bipolar arrangement with two 30 mm diameter adhesive electrodes were used for the stimulation of the EDC. Electrodes (anode and cathode) were placed over the muscle in its longitudinal plane, with one electrode at the proximal end of the muscle belly and the other electrode at the distal end of the muscle belly

**Fig. 3:**
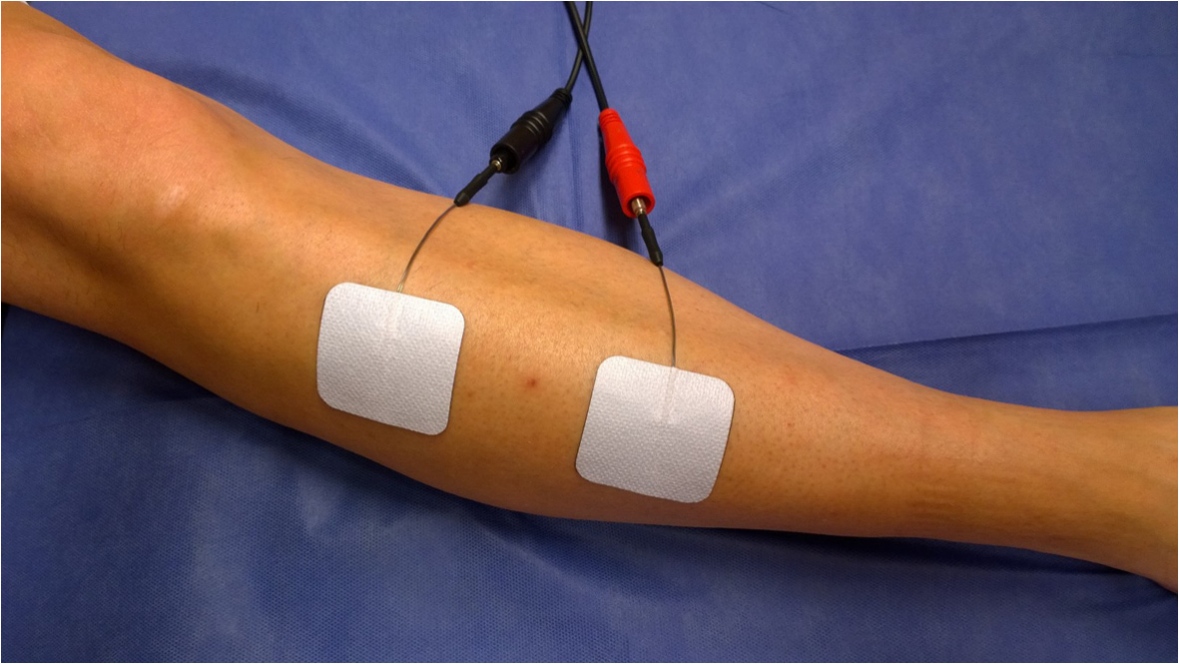
Stimulation of the tibialis anterior muscle (TA). Positioning of electrodes in bipolar arrangement with two 50×50 mm adhesive electrodes were used for the stimulation of the TA. Electrodes (anode and cathode) were placed over the muscle in its longitudinal plane, with one electrode at the proximal end of the muscle belly and the other electrode at the distal end of the muscle belly

Functional ES was applied with the constant-current electrical stimulator mentioned above. In bipolar arrangement, two 30 mm diameter adhesive electrodes (Stimcom®, Comepa, France) were used for the stimulation of the EDC (see Fig. 2) and two 50×50 mm adhesive electrodes (Stimcom®, Comepa, France) for the stimulation of the TA (see Fig. 3). Electrodes (anode and cathode) were placed over the muscle in its longitudinal plane, with one electrode at the proximal end of the muscle belly and the other electrode at the distal end of the muscle belly. The distance was adapted based on the length of the muscle. For this short evaluation surface electrodes were used as they are convenient and used in therapy. Monophasic triangular pulses were delivered to the denervated muscle to determine the threshold intensity (in mA) in order to elicit a clearly visible muscle contraction and joint movement while not causing the patient unnecessary discomfort. Stimulation of the TA induced foot dorsiflexion, stimulation of the EDC induced finger extension. We have chosen this definition of “effective muscle contraction” as it is also used in clinical practice when receiving this treatment. The current intensity was increased stepwise with 0.1 mA per second. Testing was performed with a frequency of 1 Hz in a standardized position, with a supine position for testing the TA and sitting for the EDC. Each trial for testing a combination of stimulation parameters lasted between 1 and 2 min (depending on the threshold intensity). The duration of recovery was twice the duration of one trial.

Intensity was recorded in absolute values (INT) in mA and relatively (REL) provided in percentage to the first stimulation of each patient. Due to individual differences in degree of muscle atrophy (possibly more pronounced in chronic denervation), differences in limb circumference, amount of subcutaneous fat tissue, and different skin conductance the needed absolute current intensities can vary. These individual differences can be balanced by assessing the relative measure of the applied current intensity.

Four different combinations of pulse duration and polarity were evaluated: 1) duration of 200 ms; anode proximal and cathode distal (E 200+), 2) duration of 200 ms; cathode proximal and anode distal (E 200-), 3) duration of 500 ms; anode proximal and cathode distal (E 500+) and 4) duration of 500 ms; cathode proximal and anode distal (E 500-). The sequel of ES with the different combinations of pulse duration and polarity was randomly assigned by a randomization list. The investigator who noted the muscle contraction was blinded to the applied intensity and stimulation parameters.

### Statistics

Data were analyzed using the Statistical Package for Social Sciences (SPSS) Version 15.0. Data of patients with denervated TA and denervated EDC were analyzed separately. After a check of the data’s normal distribution (testing for kurtosis and skewness, one-sample Kolmogorov-Smirnov test, *p* ≤ 0.05), a one-way within subject ANOVA was used to assess changes in INT and REL. We calculated the sphericity of the variables with the Mauchly’s test. If the condition of sphericity was not met, the inner-subject effects were tested with the Huynh-Feldt test. An alpha level of *p* ≤ 0.05 was the criterion for statistical significance. In case of statistical significance in ANOVA, planned contrast were used to test specific hypotheses on the differences between the means of the various groups.

## Results

### Denervated EDC

The Kolmogorov-Smirnov test resulted in a normal data distribution of INT and REL. Table 2 shows the descriptive data of the four different combinations of pulse duration and polarity for patients with denervated EDC. The Mauchly’s test indicated that the assumption of sphericity was violated for the main effects of INT (*χ*^2^ (5) = 25.694, *p* < 0.001) and REL (*χ*^2^ (5) = 22.205, *p* < 0.001). Therefore, we used the Huynh-Feldt test for sphericity correction to produce more accurate significant values. ANOVA did not show a significant difference between the four combinations for the two variables (Table 3).

**Table 2:** Descriptive data of the different pulse combinations for patients with denervated EDC muscle

	INT (mA)	REL (%)
E 200+	5.4 ± 2.3	105.2 ± 20.5
E 200−	5.3 ± 2.5	103.5 ± 31.7
E 500+	5.0 ± 2.4	96.0 ± 21.9
E 500−	5.6 ± 3.0	104.3 ± 29.0

Data in mean ± SD (range), *INT* intensity recorded in absolute values in mA, *REL* intensity relatively provided in percentage to the first stimulation of each patient, *EDC* extensor digitorum communis

**Table 3:** One-way within subject ANOVA of patients with denervated EDC muscle

	df	F	Level of significance
INT (mA)	2.4	0.97	0.4
REL (%)	2.3	0.99	0.4

*INT* intensity recorded in absolute values in mA, *REL* intensity relatively provided in percentage to the first stimulation of each patient, *EDC* extensor digitorum communis, *df* degrees of freedom, *F* F-ratio, level of significance (*p*-value) of the two variables

### Denervated TA

The Kolmogorov-Smirnov test resulted in a normal data distribution of INT and REL. Table 4 shows the descriptive data of the four different combinations of pulse duration and polarity for patients with denervated TA. Mauchly’s test indicated that the assumption of sphericity was violated for the main effects of INT (*χ*^2^ (5) = 22.765, *p* < 0.001) and REL (*χ*^2^ (5) = 37.108, *p* < 0.001). Therefore, we used the Huynh-Feldt test for sphericity correction to produce more accurate significant values. ANOVA evaluated a significant difference between groups for REL, and for INT a tendency to significance (see Table 5). Contrasts revealed lower values for REL, thus E 200- differs significantly from E 200+, E 500+ and E 500- (F (1,23) = 8.367, *p* = 0.008) (see Table 4).

**Table 4:** Descriptive data of the different pulse combinations for patients with denervated TA muscle

	INT (mA)	REL (%)
E 200+	8.9 ± 3.8	123.0 ± 36.1
E 200−	7.4 ± 4.2	97.6 ± 26.5
E 500+	8.5 ± 3.1	118.3 ± 35.0
E 500−	8.1 ± 4.3	108.5 ± 35.2

Data presented in mean ± SD (range), *INT* intensity recorded in absolute values in mA, *REL* intensity relatively provided in percentage to the first stimulation of each patient, *TA* tibialis anterior

**Table 5:** One-way within subject ANOVA of patients with denervated TA muscle

	df	F	Level of significance
INT (mA)	1.4	3.1	0.073
REL (%)	1.7	4.7	0.02

*INT* intensity recorded in absolute values in mA, *REL* intensity relatively provided in percentage to the first stimulation of each patient, *TA* tibialis anterior, *df* degrees of freedom, *F* F-ratio, level of significance (*p*-value) of the two variables

## Discussion

Patients with denervated TA presented significant differences in the variable REL. E 200- needed significantly less relative intensity to obtain a good visible and functional effective muscle contraction. In case of denervated EDC, the outcome variables INT and REL showed no significant differences between the different groups.

We used bipolar electrode placement as it is known to obtain higher-magnitude evoked contractions [1] and stimulates the whole muscle. The current is applied in the long axis of the muscle fibers, stimulating at each end of the muscle belly [2]. Furthermore, it is the most suitable electrode configuration in clinical practice. In the TA, the relevance of the polarity might be due to the intrinsic properties of the muscle, such as the individual pattern of motor nerve branching [21, 22], and the anatomy of the endplate zones. The TA is innervated by 2 to 4 motor nerve branches of the deep peroneal nerve [23]. It was discussed that individual motor nerve branching might influence the muscle’s response to ES [24]. Bowden et al. identified the area of the lowest electrical threshold and maximum muscle response to ES in human TA in the proximal part of the muscle [25]. The negatively charged electrode (cathode) is usually more effective in activating excitable tissue than the positively charged electrode (anode) [1, 2]. This may explain why a significantly lower intensity is needed when the cathode is placed at the proximal part of the muscle (E 200-). In contrast to the TA, the motor points are located in the middle third of the muscle in the EDC [26].

A key factor for preservation of muscle mass and function is the number of induced contractions [18, 27]. Therefore, a highly intensive stimulation protocol with long-term ES and a high number of muscle contractions is recommended [9, 11, 13]. The minimization of the amount of current applied while inducing a visible and valid muscle contraction has a high impact on acceptance by patients performing ES, especially over a longer time period. The minimizing of current intensity could reduce the sensation of discomfort, which may be the major limitation of ES [21]. Furthermore, for long time use a current of low intensity is requested in light of possible skin irritation as well as electrochemical burns. These are caused by pH changes at the electrode-tissue interface and appear if the stimulation is prolonged or with a high current density [28]. In preventing electrochemical injuries one of the most important factors beside the proper electrode type is minimizing and maintaining the current intensity as low as possible but still inducing a muscle contraction [29]. In this study, an extension of the pulse duration from 200 ms to 500 ms did not result in a decrease in intensity (see Table 4). Therefore, the use of 200 ms rather than 500 ms is preferable, since a longer pulse duration increases the risk of skin irritation by increasing the total amount of current flow. In contrast to rectangular pulses, the triangular pulse allows a selective stimulation of the denervated muscles without stimulation of the neighboring innervated muscles.

We chose the above mentioned peripheral nerve lesions as they are the most frequently transferred to our laboratory which are usually treated with ES. The radial nerve is indicated as the most frequently injured peripheral nerve in the upper extremity [30, 31] and frequently injured secondary to fractures [32]. The peroneal nerve is known to be the most sensitive peripheral nerve in the lower extremity to lesions due to poor vascularization and a superficial position [33]. Mostly stretch, contusions, lacerations, tumors, entrapments or compressions are the causes for this lesion [34]. Because the TA is the main muscle responsible for ankle dorsiflexion it is often electrically stimulated to prevent drop foot and therefore to improve locomotion efficiency. We do not think that our results can be generalised to other patient populations and muscle groups. Perhaps a longer duration after the injury or a greater denervation will have an impact on the reaction to ES. Therefore, we used REL because in patients with more chronic and more pronounced nerve lesions absolute current intensities might differ due to the greater muscle atrophy.

The visual assessment of the effective muscle contraction might be seen as limitation. The small sample size and cross-sectional design are further limitations in this study.

## Conclusions

Based on our results, we recommend ES of the denervated TA with triangular current pulses with a duration of 200 ms and a polarity with the cathode applied proximally. In case of denervated EDC, polarity and pulse duration seem to be without impact. With these optimized stimulation parameters the effect of ES with triangular pulses in peripheral nerve lesions has to be evaluated in human follow-up studies in comparison to rectangular pulses.

## Abbreviations

ES: Electrical stimulation
EDC: Extensor digitorum communis muscle
TA: Tibialiss anterior muscle
